# Determination via an Integration Strategy of the Potential Dermal ECM Reconstruction Mechanism by Which the WAW Formula Alleviates Skin Aging

**DOI:** 10.1111/jocd.70124

**Published:** 2025-03-31

**Authors:** Yingying Lin, Yunhee Chang, Yoojae Maeng, Xiaoxing Liu, Xinke Liu, Hong Meng, Fan Yi

**Affiliations:** ^1^ Beijing Key Laboratory of Plant Resources Research and Development Beijing Technology and Business University Beijing People's Republic of China; ^2^ LG H&H Co., Ltd. LG Science Park R&D Center Seoul Republic of Korea; ^3^ LG H&H Co., Ltd. China R&D Center Shanghai China

**Keywords:** ECM, integration strategy, skin aging, traditional Chinese medical formulas, WAW

## Abstract

**Background:**

Traditional Chinese medicine (TCM) treats skin conditions and delays aging using specific formulas. TCM links skin health to internal organ functions: kidney qi deficiency accelerates aging, whereas spleen deficiency leads to dry, loose skin from insufficient qi and blood. The renowned spleen‐tonifying and kidney‐nourishing formula “Wan An Wan” moisturizes the skin, but its antiageing mechanism remains unclear.

**Aims:**

The aim of this study was to investigate the development strategy and comprehensive methodological framework for incorporating TCM into cosmetics and to assess the mechanism of action and application potential of the WAW formula in mitigating skin aging.

**Results:**

In vitro experiments revealed that after optimizing the extraction of the active ingredients of the tailored WAW formula, it effectively inhibited NO production and tyrosinase activity and increased filaggrin, AQP, and HAS2 mRNA expression. Moreover, the active ingredients, including morroniside, were also analyzed separately. Mechanistic studies revealed that the tailored WAW formula upregulated the expression of the TGF‐β1 gene associated with ECM production and downregulated the expression of the MMP‐2/MMP‐9 genes associated with ECM degradation, regulating cell behaviors such as migration, adhesion, and proliferation and thereby maintaining the healthy state of the skin.

**Conclusions:**

In this study, an overall strategy for applying TCM for the development of skin care solutions was established, the potential value of WAW in modern dermatologic applications was revealed, innovative raw materials with comprehensive effects were developed, and new ideas for the fields of cosmetics and dermatology were provided.

**Abbreviations**
AGEs: advanced glycosylation end products
AQP3: aquaporin 3
CML: Nε‐(carboxymethyl)‐L‐lysine
COL1: collagen I
ECM: extracellular matrix
FN1: fibronectin I
HAS2: hyaluronan synthase 2
LM5: laminin 5
MGO: pyruvaldehyde
MMP‐2: matrix metalloproteinase‐2
MMP‐9: matrix metalloproteinase‐9
TCM: traditional Chinese medicine
TGF‐β1: transforming growth factor‐β1
TNC: tenascin C
WAW: Wan An Wan

## 1 Introduction

Traditional Chinese medicine (TCM) skincare and beauty refer to the rational application of Chinese Materia Medica to treat skin diseases, delay aging, maintain a youthful appearance, or prevent and treat cosmetic disorders. According to TCM theory, skin physiology is closely related to the functions of internal organs and related nutrient availability. For example, skin aging is believed to be accelerated by kidney deficiency, which leads to wrinkles and diminished skin vitality. Spleen deficiency can also result in deficiencies in both vital energy and blood, causing skin dryness and laxity. The famous Chinese medicinal for tonifying the spleen and kidney, “Wan An Wan,” was originally documented in the Tang Dynasty by the renowned “Medicine King” Sun Simiao in his work “Emergency Formulas Worth a Thousand in Gold—Volume Nineteen”. It is also recorded in ancient medical texts such as “Secret Essentials from the Imperial Library”, “Complete Record of Holy Relief”, and “Imperial Medical Bureau Formula”. Among these, “Complete Record of Holy Relief” renamed “Wubi Shuyu Wan” to “Cistanche Wan”, and “Imperial Medical Bureau Formula” renamed “Cistanche Wan” to “Wan An Wan”. The formula consists of *Cistanches Herba*, *Dioscorea polystachya* Turcz., *Schisandra chinensis* (Turcz.) Baill., *Eucommia ulmoides* Oliv., *Achyranthes bidentata* Blume, *Cuscuta chinensis* Lam., *Poria cocos* (Schw.) Wolf., *Alisma orientalis* (Sam.) Juz., *Halloysitum Rubrum*, *Rehmannia glutinosa* (Gaertn.) Libosch. ex Fisch. et Mey., *Cornus officinalis* Sieb. et Zucc. and *Morinda officinalis* F. C. How. The formula description states that “after seven days of use, it promotes health, moisturises the body, reddens the lips, warms the hands and feet, and brightens the face,” indicating its potential to improve skin appearance and delay aging. Modern pharmacological studies have shown that these herbs contain various bioactive compounds that possess antiageing [1], whitening [2], anti‐inflammatory [3], and moisturizing [4] activities. However, the overall effect of the formula on skin aging has not been extensively documented, requiring further exploration.

However, the topical application of TCM formulas to the skin raises concerns regarding the adherence of herbal ingredients to modern medical theories and the safety of these formulas, as well as issues related to product color and odor and other factors. Moreover, TCM compounds act on multiple molecular targets or biochemical pathways, which complicates the ability of traditional experimental studies to elucidate the comprehensive mechanisms by which formulas such as WAW affect skin aging. TCM compounding theory presents a framework to effectively tailor Chinese herbal formulas, re‐compound herbs, construct systematic networks to explore potential molecular mechanisms, and validate them through cellular experiments, providing a new strategy for investigating the complex components and mechanisms of Chinese medicines. This method has numerous advantages and has been widely applied in the study of active ingredients, activity targets, and therapeutic functions in Chinese medicine.

Therefore, in this study, WAW was first tailored to adapt to modern needs and standards for slowing skin aging. A preliminary investigation of the effects of the medicinal substances in tailored WAW on the skin was carried out by measuring parameters such as the collagen content, tyrosinase activity, and NO production. The active compounds, their targets, and their predicted pathways were integrated using network pharmacology methods, and the extracted active ingredients were optimized to enhance the formula's effect. Then, based on the TCM compounding theory, in vitro biochemical experiments and network pharmacology results, the network pharmacology of the tailored formula was optimized to reduce side effects and optimize efficacy. The chemical constituents of the formulas screened for better overall efficacy were subsequently identified via HPLC. To validate the bioinformatics results, cellular‐level experiments were conducted to evaluate the pharmacological effects of the tailored WAW formula and to elucidate the mechanisms of the aging‐delaying effect of WAW to increase its scientific and practical application value.

## 2 Materials and Methods

### 2.1 Materials

The medicinal substances in the WAW formula, *D. polystachya* , *C. chinensis* , *C. officinalis* , *S. chinensis* , *E. ulmoides* , *A. bidentata* , *P. cocos* , and *A. orientalis* , were purchased in 2021 from Shanghai Yanghetang Traditional Chinese Medicine Drinking Tablets Co.

### 2.2 Methods

#### 2.2.1 Modification of the WAW Formula

The original WAW formula consists of *C Herba*, *D. polystachya* , *S. chinensis* , *E. ulmoides* , *A. bidentata* , *C. chinensis* , *P. cocos* , *A. orientalis* , *H Rubrum*, *R. glutinosa* , *C. officinalis* , and *M. officinalis* . This prescription was tailored to meet the needs of external use, such as color and texture considerations. The names of the medicinal substances included in the formula were standardized according to the 2015 Pharmacopeia of the People's Republic of China and the nomenclature conventions for modern Chinese medicine studies. Any drug components not included in the pharmacopeia could not be entered and systematically analyzed and thus were excluded. The formulas were constructed according to the interactions between medicinal substances and the laws of compounding, as well as factors such as the nature and attributes of the medicinal substances and the requirements of modern skincare products.

#### 2.2.2 In Vitro Experiments to Explore the Effects of the Tailored WAW Formula on Skin

Each medicinal substance was extracted according to the design of the experimental programme, changing as necessary the factors and parameters of the extraction process conditions and using ultrasound‐assisted extraction and then reduced‐pressure extraction and filtration to generate 10 g of each substance. The eight medicinal substances were evaluated for collagen content, NO content, tyrosinase activity, and melanin content to determine whether these substances had any effect on the skin and to perform a preliminary exploration to a provide research direction for subsequent experiments.

##### 2.2.2.1 Griess Method for Determining the NO Content

Fibroblasts were seeded at a concentration of 2 × 10^5^ cells/ml in 24‐well plates and incubated for 24 h. After the medium was removed, the cells were cultured in serum‐free medium for 12 h. After 30 min of treatment, the cells from the control group (treated with serum‐free medium), sample group (treated with the eight extracted medicinal substances), and positive control group (treated with 100 μM L‐NMMA) were then treated with 500 ng/mL LPS for 18 h. After incubation, the supernatant was transferred to a 96‐well plate, Griess reagent was added, and the reaction was allowed to proceed for 15 min at room temperature. The absorbance at 540 nm was measured using an ELISA reader.

##### 2.2.2.2 Tyrosinase Activity and Melanin Content Measurement

Tyrosinase activity and melanin content were measured in MNT‐1 human melanoma cells. The cells were seeded at 2 × 10^5^ cells/ml in 6‐well plates and incubated for 24 h. After 72 h of treatment, the cells in the control group (treated with serum‐free medium), sample group (treated with the eight extracted medicinal substances), and positive control group (treated with 200 μg/mL arbutin) were harvested. The cells were then lysed via centrifugation at 13000 rpm for 1 min, the supernatant was removed, and 300 μL of 0.5% Triton X‐100 solution was added. The mixture was centrifuged again at 13000 rpm for 3 min, and the pellet and supernatant were collected separately. The pellet was incubated with 100 μL of 0.5 N NaOH overnight to release melanin, and the absorbance at 450 nm was measured using an ELISA reader. To measure the tyrosinase activity, 100 μL of the cell lysate supernatant was mixed with 1 mM L‐DOPA solution and incubated at 37°C for 1 h. The absorbance at 450 nm was measured using an ELISA reader.

#### 2.2.3 Network Pharmacological Analysis

##### 2.2.3.1 Collection of Compounds and Target Prediction for the Tailored WAW Formula

The structures of the compounds distributed in the tailored WAW formula were collected from the Traditional Chinese Medicine Systems Pharmacology Database and Analysis Platform (https://tcmsp‐e.com/tcmsp.php, TCMSP), plotted with ChemOffice, and converted into three‐dimensional structure files (mol2 format) using Open Babel software to construct a database of compound information for the plant compositions. Discovery Studio (version DS 2020) was used to predict the targets of the active ingredients of the tailored WAW formula through virtual docking, and candidate targets of WAW were also identified. The genes corresponding to the obtained targets were defined using PDB, UniProt, and DAVID.

##### 2.2.3.2 Construction of a Medicinal Substance‐Active Compound‐Target Network

The GeneCards (Human Gene Database, https://www.genecards.org/) and NCBI Gene (http://www.ncbi.nlm.nih.gov/gene/) databases were screened for skin‐related genes, and the results of both sets were concatenated to obtain the set of skin‐related gene targets. A Venn diagram was used to identify overlaps between the skin‐related targets and potential targets of the tailored WAW formula.

Search Tool for the Retrieval of Interacting Genes (STRING, https://cn.string‐db.org) was used to investigate the interactions among these protein‐coding genes with a confidence level greater than 0.4 [5]. The protein–protein interaction (PPI) network and related network data files were obtained by importing the intersecting targets in the STRING database. The network data files were imported into Cytoscape to establish the PPI networks. The NetworkAnalyzer module was used to visualize the network and obtain the degree and other information. The drug–compound–target network was constructed using Cytoscape.

##### 2.2.3.3 Gene Ontology (GO) and Kyoto Encyclopedia of Genes and Genomes (KEGG) Enrichment Analyses

Considering the overall action of the formula, the UniProt genes of the eight medicinal substances were combined, and the obtained potential targets were subjected to Kyoto Encyclopedia of Genes and Genomes (KEGG) signaling pathway enrichment analysis and Gene Ontology (GO) bioprocess enrichment analysis in the human gene annotation database DAVID 6.8 (https://david.ncifcrf.gov/). The target genes were screened for *p* < 0.05, and the pathways and biological processes associated with the actions of the tailored WAW formula were analyzed.

#### 2.2.4 Optimisation of the Extracted Individual Medicinal Substances in the Tailored WAW Formula

##### 2.2.4.1 One‐Way Experimental Methods

Network pharmacology and molecular docking analyses were used to predict the main active ingredients of the tailored WAW formula, mainly iridoids, lignans, flavonoids, etc., and then to optimize the process for extracting the main ingredients. We weighed 10 g of powdered medicinal substances and investigated the effects of the liquid‐to‐material ratio, ultrasonication time, and extraction time on the flavonoid, saponin, lignan, and triterpene contents of different medicinal substances. The results were collected under the following experimental conditions: ultrasonication time—60 min, 1,3‐butanediol—30%, number of extractions—1, liquid/feed ratios—1:10, 1:20, 1:30, 1:40, and 1:50 (g/ml); ultrasonication times—40, 50, 60, 70, and 80 min, 1,3‐butanediol—30%, number of extractions—1, liquid/feed ratio—1:20 (g/ml); and ultrasonication time—60 min, 1,3‐butanediol—30%, number of extractions—1, 2, 3, and 4, liquid/feed ratio—1:20 (g/ml). The above experiments were repeated three times in parallel for each group, and the results were averaged.

##### 2.2.4.2 Orthogonal Experimental Methodology

According to the results presented in section 2.2.4.1, the number of extractions for each substance was as follows: *D. polystachya* , 2; *C. chinensis* , 2; *C. officinalis* , 3; *S. chinensis* , 2; *E. ulmoides* , 3; *A. bidentata* , 3; *A. orientalis* , 3; and *P. cocos* , 3. The orthogonal experimental factor level design is shown in Table 1. The contents of saponins, flavonoids, triterpenes, and lignans in the extracts of the different medicinal substances were detected to analyze and optimize the experimental results.

**TABLE 1:** Orthogonal experimental factor level design.

Medicinal substance	Level	A: Material‐liquid ratio (g/ml)	B: Ultrasonic time (min)
*D. polystachya*	1	1:20	60
	2	1:30	70
*C. chinensis*	1	1:20	60
	2	1:30	70
*C. officinalis*	1	1:20	70
	2	1:30	80
*S. chinensis*	1	1:20	70
	2	1:30	80
*E. ulmoides*	1	1:10	60
	2	1:20	70
*A. bidentata*	1	1:20	50
	2	1:30	60
*P. Cocos*	1	1:20	70
	2	1:30	80
*A. orientalis*	1	1:20	60
	2	1:30	70

#### 2.2.5 Determination of Optimal Formulas and Characterization of Components by HPLC

Based on the principles of traditional Chinese medicine prescriptions, the results of in vitro efficacy experiments of single herbs, and the network pharmacology results, the combinations and ratios of the single herbs in the WAW formula were adjusted, resulting in three different formulation ratios: M1, M2, and M3 (Table 8). The three formulas were subsequently subjected to anti‐inflammatory evaluation (NO inhibition assay and whitening evaluation), tyrosinase activity measurement, and moisturizing evaluation (filaggrin, AQP3, and HAS2 gene expression level measurements as described in section 2.2.6.4) to identify the most effective formulas to undergo subsequent identification of the components by HPLC and cellular assay validation.

After the compounding ratio was determined, the quality markers of the formulas were studied using HPLC. The characteristic components of the single herbs were selected to optimize the chromatographic conditions. The qualitative analyses were carried out by retention time and ultraviolet spectrograms under suitable chromatographic conditions.

High‐performance liquid chromatography (HPLC) parameters: The chromatographic column used was Poroshell HPH‐C18 (3 × 100 mm, 2.7 μm); the mobile phase was water:acetonitrile (99:1); the flow rate was 0.8 mL/min; the detection wavelengths for morroniside, loganin, schisandrin A, pinoresinol diglucoside, and β‐ecdysterone were 240 nm, 238 nm, 215 nm, 200 nm, and 248 nm, respectively; the column temperature was 30°C; the detector was a diode array detector with an injection volume of 20 μL; and the gradient elution was 0~25 min, 95%–0% A, 5%–100% B; 25~30 min, 100% B; 30~35 min, 0%–95% A, 100%–5% B; and 35~37 min, 95% A, 5% B (A: acetonitrile, B: water).

#### 2.2.6 Validation of the Efficacy of MGO‐Induced Cellular Senescence Modeling

##### 2.2.6.1 Modeling of Cell Senescence Damage

The MGO stimulation‐induced fibroblast senescence model was established by Liu Xiaoxing [6]. The CCK‐8 assay was used to measure the cell survival rate after different concentrations of MGO solution (W296902, Sigma‐Aldrich, USA) were used to stimulate the cells for 48 h. The CCK‐8 reagent (BN15201, BiOrigin, USA) was mixed with serum‐free DMEM at a ratio of 1:10, and 110 μL of the mixture was added to each well. After incubation at 37°C in the dark for 1 h, the absorbance at 450 nm (OD value) was measured with an enzyme counter to calculate the cell viability (%).

$$\text{Cell viability}\;\left(\%\right)=\frac{{\mathrm{OD}}_{\text{Sample}}}{{\mathrm{OD}}_{\text{Control}}}\times 100$$

After the cells were stimulated with different concentrations of MGO for 48 h in 6‐well plates, 200 μL of cell lysate was added to each well, and the plates were centrifuged at 10000 rpm for 2 min at 4°C. The supernatant was extracted using a BCA protein quantification kit (P0010, Beyotime, China) and a human CML ELISA kit (CSB‐E12798h, Cusabio, China) to determine the expression of CML.

$$\mathrm{CML}\;\text{exression level}\;\left(\mathrm{pg}/\mathrm{\mu g}\right)=\frac{\mathrm{CML}\;\text{conent}\;\left(\mathrm{pg}/\mathrm{mL}\right)}{\text{Total protein content}\;\left(\mathrm{\mu g}/\mathrm{mL}\right)}$$

##### 2.2.6.2 Cellular Behavioral Dysfunction Detection

The cell viability assay to measure the cytotoxic effects of formula M3 and L‐myostatin was performed as described in section 2.2.6.1.

The cell proliferation capacity was assessed using the CCK‐8 method. Human skin fibroblasts were cultured in a 96‐well plate, and these fibroblasts were divided into 4 groups: control group (treated with serum‐free culture medium), MGO model group (treated with 4 mmol/L MGO solution), MGO + M3 group (treated with 4 mmol/L MGO and varying concentrations of M3 for coculture), and MGO + L‐carnitine (positive control) group (treated with 4 mmol/L MGO and varying concentrations of L‐carnitine for coculture), followed by the same methods described in section 2.2.6.1. Four groups of reagents were added to a 24‐well plate covered with cells and cultured for 48 h, and the cell adhesion rate was determined using a Cell Adhesion Assay Kit (BB‐48123, China Biosbio Company). Fluorescence microscopy images were captured, and ImageJ software was used to quantify adherent cells. A straight line was drawn across each well in a 24‐well plate seeded with cells using a 200 μL pipette tip. The corresponding reagents were added to the control group, MGO model group, MGO + M3 group, and positive control group (L‐carnosine), and the cells were further cultured for 24 and 48 h, followed by observation and imaging under a microscope. ImageJ software was used to calculate the percentage of scratch area recovery. The calculation method for the scratch area recovery rate was as follows:

$$\begin{array}{c} \text{Scratch area recovery ratio}\;\left(\%\right)=\;\\ \frac{0\;h\;\text{Scratch area}-24/48\;h\;\text{Scratch area}}{0\;h\;\text{Scratch area}} \end{array}$$

##### 2.2.6.3 Detection of the ECM Protein Content by ELISA

The supernatants of the cells in the four treatment groups were collected after 48 h. The protein contents in the cell culture supernatants were detected using a COL1 detection kit (SEA571Hu, Yunclone, China), FN1 detection kit (SEA037Hu, Yunclone, China), LM5 detection kit (SEC078Hu, Yunclone, China) and TNC detection kit (SEB975Hu, Yunclone, China).

##### 2.2.6.4 Real‐Time Fluorescence Quantitative PCR (qRT–PCR) Analysis

Different HFF‐1 groups were incubated in 6‐well plates following treatment. The RNA was extracted via the TRIzol method, and the first‐strand cDNA was synthesized using the UEIris II RT‐PCR system (BN12028, Biotek, China). Fast Super EvaGreen qPCR Master Mix (BN12008, Biotek, China) was used for quantitative PCR (qPCR) analysis. The primers used in the present study were designed using the NCBI platform (www.ncbi.nlm.nih.gov), as detailed in Table 2.

**TABLE 2:** Primer sequences.

Primer	Primer sequence
GAPDH‐F	TGCACCACCAACTGCTTAGC
GAPDH‐R	GGCATGGACTGTGGTCATGAG
Filaggrin‐F	CGAAGGAGCCAAAAATATAAAACAG
Filaggrin‐R	GATGTGCTAGCCCTGATGTTGA
AQP3‐F	ACCTTTGCCATGTGCTTCCT
AQP3‐R	GCGTCTGTGCCAGGGTGTA
HAS2‐F	CCCAAAATGTGAAGCTTGGT
HAS2‐R	CAGGCCACAGAACAAAACCT
GAPDH‐F	ACCACTTTGTCAAGCTCATT
GAPDH‐R	GTGAGGGTCTCTCTCTTCCT
TGF‐β1‐F	CAATTCCTGGCGATACCTCAG
TGF‐β1‐R	GCACAACTCCGGTGACATCAA
MMP2‐F	GTCTGTGTTGTCCAGAGGCA
MMP2‐R	CTAGGCCAGCTGGTTGGTTC
MMP9‐F	CTTTGAGTCCGGTGGACGAT
MMP9‐R	TCGCCAGTACTTCCCATCCT

### 2.3 Statistical Analysis

Three parallel experiments were performed for all of the above experiments, and the results are expressed as the means ± standard deviations. The data were visualized and statistically analyzed using GraphPad Prism 9 and ImageJ, and the significance of differences was calculated using one‐way ANOVA and two‐way ANOVA. (Note: #*P* < 0.05, ##*p* < 0.01, ###*p* < 0.001 and #### *p* < 0.0001 compared with the control group. *p < 0.05, **p < 0.01, ***p < 0.001 and ****p < 0.0001 compared with the MGO model group).

## 3 Results

### 3.1 Preliminary Study on the Effects of the Tailored WAW Formula on Skin

#### 3.1.1 Effects of the WAW Formula After Addition or Subtraction of Substances

Experts in traditional Chinese medicine pharmacology, theory, and modern research, as well as in the development of botanical ingredients for cosmetics, have analyzed and explored the components of the classic herbal formula “Wan An Wan” (WAW). In accordance with the principles of herb interaction, compatibility, flavor, and meridian tropism and considering the requirements of modern skincare products, the tailored WAW formula has been reformulated to include *D. polystachya* , *C. chinensis* , *C. officinalis* , *S. chinensis* , *E. ulmoides* , *A. bidentata* , *A. orientalis* , and *P. Cocos* .

Among these, *D. polystachya* and *C. chinensis* are the sovereign medicinal agents. *D. polystachya* , with its sweet flavor and neutral nature, moisturizes and nourishes the lungs and tonifies the kidneys to secure essence. *C. chinensis* , known since “Shennong's Classic of Materia Medica,” has a spicy‐sweet flavor and neutral nature and is moisturizing and nourishing. From the perspective of traditional Chinese medicine, the nourishment and circulation of blood and essential maintenance are closely related to skin health and the promotion of natural, radiant skin.

The minister medicines among the components of WAW are *C. officinalis* and *S. chinensis* . *C. officinalis* tastes sour and slightly warm, with astringent properties to consolidate and stop leakage. *S. chinensis* tastes sour–sweet and is warm, with astringent properties to contract and solidify. Both are sour medicines with astringent and solidifying properties, which are beneficial for dry and flaking skin and maintaining facial stability. With abundant essence, qi, blood, and body fluids promoting flourishing and facial radiance, *C. officinalis* and *S. chinensis* can consolidate essence, thereby nourishing the skin.

The assistant medicines in WAW are *E. ulmoides* and *A. bidentata* . *E. ulmoides* tastes sweet and warm, tonifies yang, and nourishes essence. *A. bidentata* tastes bitter‐sweet and is neutral, promoting blood circulation and alleviating stasis.

The courier medicines are *P. cocos* and *A. orientalis* , which promote fluid production and clear turbidity. *P. cocos* tastes sweet and bland, with a neutral nature, promoting diuresis and dampness dispersion, whereas *A. orientalis* tastes sweet and bland, with a cold nature, promoting diuresis, dampness dispersion, heat clearing, and turbidity transformation. Both *P. cocos* and *A. orientalis* belong to the category of sweet and bland medicines.

#### 3.1.2 Results of In Vitro Efficacy Tests

**FIGURE 1:**
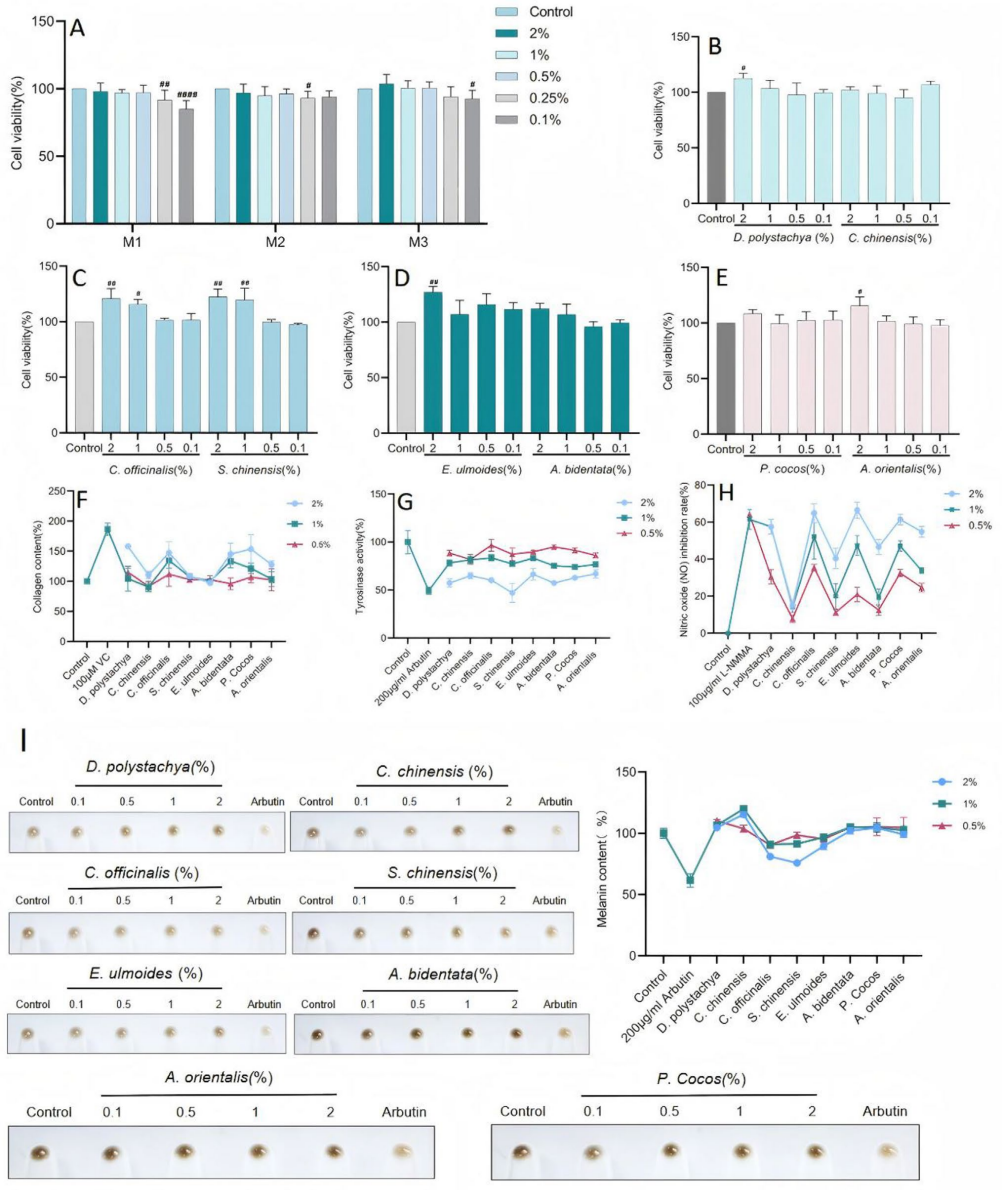
(A) Cytotoxicity assay of three formulas with different compound ratios; (B) *D. polystachya* and *C. chinensis* cytotoxicity assay; (C) *C. officinalis* and *S. chinensis* cytotoxicity assay; (D) *E. ulmoides* and *A. bidentata* cytotoxicity assay; (E) *P. cocos* and *A. orientalis* cytotoxicity assay; (F) Collagen content assay; (G) Tyrosinase activity assay; (H) NO assay; (I) Effects of single herbs on the pigmentation of MNT‐1 melanoma cells. (The results are expressed as the means ± SD. One‐way ANOVA and Two‐way ANOVA was performed to determine statistical significance. #*P* < 0.05, ##*P* < 0.01, ###*P* < 0.001 and ####*P* < 0.0001 compared to the control group).

The three different groupings of compound ratios were nontoxic to cells at concentrations of 0.1%–2% (Figure 1A), and the eight individual herbs were nontoxic to cells at concentrations of 0.5%–2% (Figure 1B–E); therefore, overall concentrations of 2%, 1%, and 0.5% were selected for the subsequent experiments.

To assess the potential skin health benefits of the eight herbs in the tailored WAW formula and to understand their effects on skin improvement from a modern perspective, we evaluated the eight herbs of WAW in terms of their ability to increase collagen synthesis in the skin, which in turn improves skin firmness and reduces fine lines; to effectively modulate tyrosinase activity to reduce hyperpigmentation or blotchiness; and to regulate nitric oxide levels to improve skin and reduce inflammation. The experimental results (Figure 1F–I) revealed that the samples, when applied at 2% concentrations, ranked in terms of collagen content as follows: *D. polystachya* > *P. cocos* > *C. officinalis* > *A. bidentata* ; the other herbs did not promote collagen. They ranked in terms of inhibition of tyrosinase activity as follows: *S. chinensis* > *A. bidentata* > *D. polystachya* > *C. officinalis* > *P. cocos* > *C. chinensis* > *E. ulmoides* > *A. orientalis* . They ranked in terms of inhibition of NO production as follows: *E. ulmoides* > *C. officinalis* > *P. cocos* > *D. polystachya* > *A. orientalis* > *C. officinalis* > *S. chinensis* > *C. chinensis* . They ranked in terms of melanin inhibition as follows: *S. chinensis* > *C. officinalis* > *E. ulmoides* ; other herbs did not have a melanin‐inhibiting effect. When applied at a 1% concentration, *C. officinalis* and *A. bidentata* produced some collagen synthesis‐promoting effects, whereas the other herbs did not significantly promote collagen synthesis. *S. chinensis, A. orientalis*, *A. bidentata* , and *P. cocos* had certain effects on inhibitory tyrosinase activity. *D. polystachya* and *C. officinalis* better inhibited NO production. Only *C. officinalis* and *S. chinensis* had some inhibitory effects on melanin synthesis. When applied at a concentration of 0.5%, the eight herbs had no obvious effect on promoting collagen synthesis or inhibiting melanin; only *C. chinensis* inhibited tyrosinase activity; *D. polystachya* and *C. officinalis* inhibited the production of NO better; and only *C. officinalis* had some inhibitory effects on melanin synthesis.

### 3.2 Analysis of the Composition of the Tailored WAW Formula and Prediction of the Mechanism of Action for Slowing Skin Aging

#### 3.2.1 Drug‐Active Ingredient‐Target Network

In the search box of the TCMSP homepage, search for the eight medicinal herbs identified [7], as shown in Table 3. The number of compounds for each herb was as follows: *D. polystachya* (71 compounds), *C. chinensis* (29 compounds), *C. officinalis* (226 compounds), *S. chinensis* (130 compounds), *E. ulmoides* (147 compounds), *A. bidentata* (176 compounds), *P. cocos* (34 compounds), and *A. orientalis* (46 compounds).

**TABLE 3:** Summary of the results of the TCMSP search for the components of the tailored WAW formula.

TCMSP No.	Latin name	Number of compounds
359	*D. polystachya*	71
407	*C. chinensis*	29
361	*C. officinalis*	226
41	*S. chinensis*	130
114	*E. ulmoides*	147
304	*A. bidentata*	176
129	*P. Cocos*	34
480	*A. orientalis*	46

**FIGURE 2:**
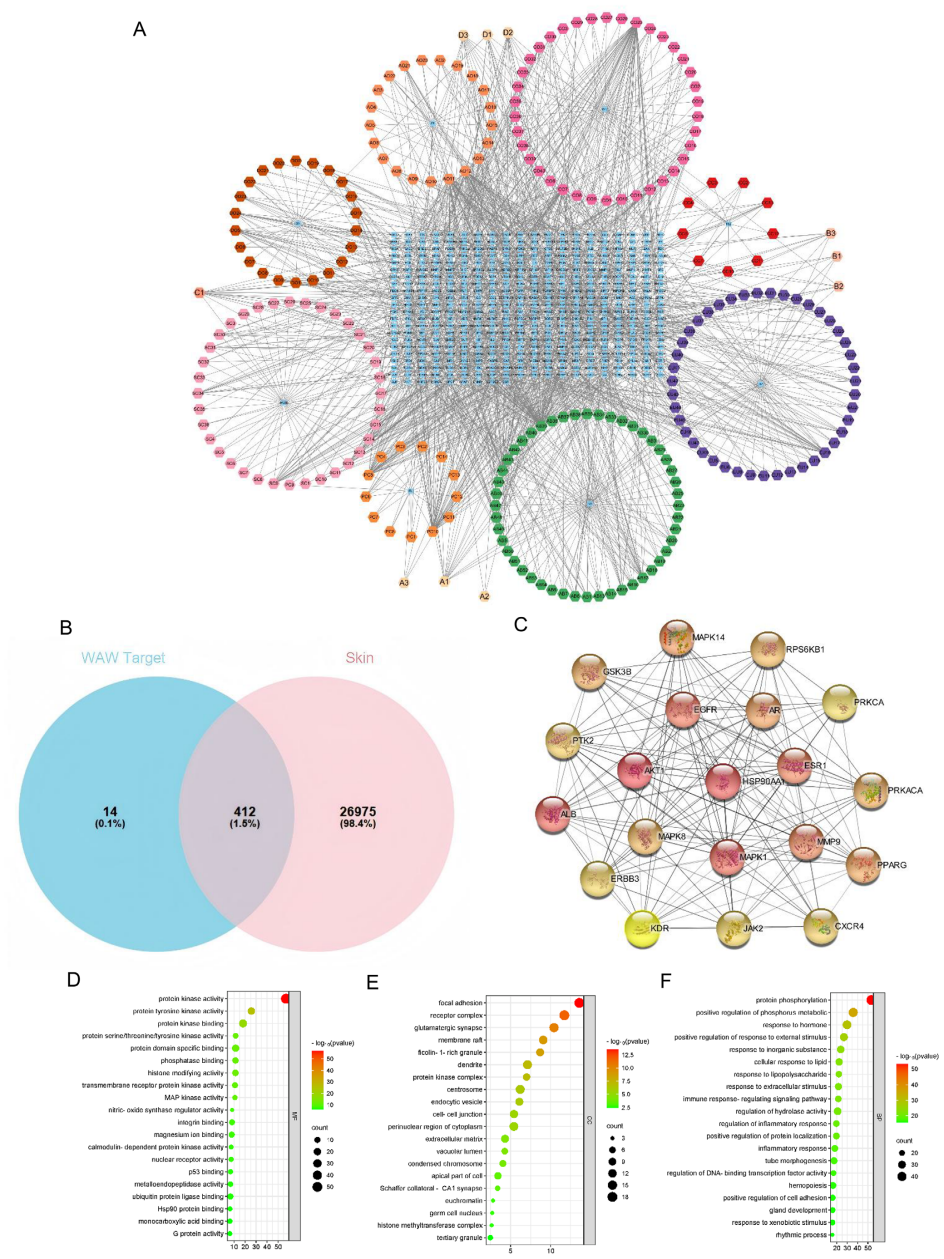
(A) Drug‐active ingredient‐target network process for discovering key crossover genes; (B) Venn diagram of the tailored WAW formula with skin‐related genes; (C) Top 20 PPI networks and network degree values for the tailored WAW formula with skin‐related targets; (D–F) Gene Ontology (GO) enrichment analysis. The X‐axis represents the enrichment rate of these genes among the total genes, whereas the Y‐axis represents the enrichment pathways of the target genes (*p* value < 0.05). The depth of the color represents the magnitude of the value, and the size of the circle represents the enrichment counts of these pathways.

Using Cytoscape 3.9.0 software, the drug‐active ingredient‐target network of the tailored WAW formula was constructed (Figure 2A), with blue rectangles representing the 412 action targets, hexagons representing the active ingredients of the 8 single medicinal substances, pale yellow hexagons representing the common active ingredients of the herbs, and each edge representing the interaction between the drug, the active ingredient, and the target. This regulatory network had a total of 825 nodes, 9805 edges, and an average degree value of 15.084. Core node screening was performed based on the network topological features such as the node degree value, and the information table of the 10 active ingredients with the highest degree values in the specific drug‐active ingredient‐target‐of‐action network is shown in Table 4.

**TABLE 4:** Information on the active ingredients with the top 10° values in the drug‐active ingredient‐target network.

MOL ID	Name	Category	Degree
MOL012516	Geniposide	Iridoid	382
MOL012524	Hmp‐hmpep	Alkaloid	252
MOL004367	Olivil	Lignan	214
MOL000841	Raffinose	Polysaccharide	156
MOL000838	Alpha‐D‐Galp‐(1‐ > 6)‐alpha‐D‐Galp‐(1‐ > 6)‐D‐Glu	Polysaccharide	144
MOL007228	Kaempferol‐3‐o‐beta‐d‐glucoside	Flavonoid	134
MOL006126	Hexahydrocurcumin	Iridoid	119
MOL000173	Wogonin	Flavonoid	105
MOL000278	Beta‐glucan	Polysaccharide	104
MOL001680	Loganin	Iridoid	101

The main active ingredients of the tailored WAW formula, as determined by network pharmacological research methods, are cyclic enol ether terpenoids, alkaloids, lignans, polysaccharides, flavonoids, etc. These findings provide suggestions and references for further research.

#### 3.2.2 KEGG Pathway and GO Enrichment Analysis

We obtained 23 747 and 15 094 potential target genes from the GeneCards and NCBI Gene databases, respectively, with the search terms “Skin/Skin disease/dermatology” and “ *Homo sapiens* ”, respectively, and 27 387 skin‐related target genes were obtained after duplicates. By intersecting the active ingredient regulatory targets of the tailored WAW formula with skin‐related target genes, 412 skin‐related active ingredient targets of the tailored WAW formula were obtained (Figure 2B).

The 412 skin‐related targets of the tailored WAW formula active ingredient action targets were subjected to PPI analysis via the STRING platform, and the results were imported into Cytoscape 3.9.0 for network topology analysis. In this analysis, nodes denote the target genes, degree values denote the number of lines connected to the nodes to assess the importance of each node in the network, and each edge denotes the interaction between the target relationships. The top 20 targets are shown in Figure 2C.

To gain insight into the functions of these protein targets, we performed functional enrichment analysis using the GO and KEGG databases. After the targets of all eight drugs were combined, 429 genes were subjected to KEGG and GO signaling pathway enrichment. A total of 8 KEGG pathways (*p < 0.05*) and 13 GO entries (*p < 0.05*) associated with skin function were screened, and the main biological processes associated with skin aging are shown in Tables 5 and 6. The results revealed that the MAPK signaling pathway, the PI3K–Akt signaling pathway, and the collagen catabolic process were related to skin aging, with the related genes being TGFB, MMPs, MAPKs, etc.

**TABLE 5:** KEGG signaling pathway of the tailored WAW formula.

Number	Signaling pathways	Gene count	*p*	Gene symbol
1	VEGF signaling pathway	24	1.97E‐15	PIK3CG, RAC2, MAPK1, KDR,RAC, MAPK3, MAPK12, HRAS, MAPKAPK2, MAPK14, CDC42, PRKCA, AKT1, PTGS2, PRKCB, SPHK1, MAPKAPK3, MAPK13, MAP2K1, PTK2, RAC1, SRC, KRAS, KRAS
2	MAPK signaling pathway	38	1.02E‐09	TGFBR1, RAC2, TGFBR2, RAC3, HRAS, MAPKAPK2, RPS6KA1, MAP3K14, BRAF, MAPK14, HSPA8, MAPK8, CDC42, PRKCA, PRKCB, RAC1, EGFR, MAPK1, MAPK3, MAPK12, PAK1, HSPA6, FGFR2, RPS6KA5, FGFR1, MAP2K6, NTRK2, RAP1A, HSPA1A, PRKACA, AKT1, ZAK, MAPK10, MAPKAPK3, MAPK13, MAP3K5, MAP2K1, KRAS
3	PI3K‐Akt signaling pathway	43	2.04E‐08	RPS6KB1, SYK, RXRA, PRKAA2, MET, HRAS, HSP90AA1, CDK6, IGF1R, MDM2, JAK1, CSF1R, KIT, JAK2, IL2, CDK2, JAK3, FLT1, ITGA2B, PRKCA, SGK1, RHEB, ITGA5, RAC1, EGFR, KRAS, PIK3CG, TEK, MAPK1, KDR, MAPK3, FGFR2, FGFR1, PDPK1, GSK3B, HSP90AB1, AKT1, PCK1, INSR, EPHA2, MAP2K1, PTK2, KRAS
4	HIF‐1 signaling pathway	19	6.08E‐07	PIK3CG, TEK, RPS6KB1, MAPK1, MAPK3, IGF1R, CREBBP, FLT1, PRKCA, AKT1, PRKCB, INSR, CAMK2A, MAP2K1, TCEB2, CAMK2B, EGFR, KRAS, PFKFB3
5	Melanoma	14	2.97E‐05	PIK3CG, MAPK1, MAPK3, MET, HRAS, CDK6, FGFR1, IGF1R, MDM2, BRAF, AKT1, MAP2K1, EGFR, KRAS
6	NF‐κ B signaling pathway	12	0.00305996	CSNK2A2, XIAP, CSNK2A1, SYK, LYN, IRAK4, PRKCQ, ZAP70, PTGS2, PLAU, LCK, MAP3K14
7	Bacterial invasion of epithelial cells	11	0.004216661	PIK3CG, RHOA, ILK, CLTC, MET, CDC42, ITGA5, PTK2, RAC1, SRC, DNM1
8	Circadian entrainment	12	0.006045236	PRKG1, MAPK1, MAPK3, PRKACA, GNAI1, PRKCA, GRIA2, PRKCB, ADCY10, RPS6KA5, CAMK2A, CAMK2B

**TABLE 6:** GO signaling pathways affected by the tailored WAW formula.

Number	Signaling pathways	Gene count	*P*	Gene symbol
1	GO:0007173 ~ epidermal growth factor receptor signaling pathway	12	1.19E‐07	CSK, ABL1, HRAS, FES, PTK2B, RPS6KA5, PDPK1, PTK2, ADAM17, SRC, EGFR, KRAS
2	GO:1901796 ~ regulation of signal transduction by p53 class mediator	16	5.15E‐07	AURKB, AURKA, PRKAA2, PIN1, CDK5, MDM2, CSNK2A2, CSNK2A1, CDK2, BLM, PRMT5, MAPK14, CHEK1, EHMT1, AKT1, CHEK2
3	GO:0043627 ~ response to estrogen	11	4.70E‐06	TEK, CA2, MAPK1, LDHA, TGFBR2, F7, ESR1, HSP90AA1, PPARG, GBA, KMT2D
4	GO:0007568 ~ aging	15	7.42E‐05	MMP7, AURKB, TYMS, RPS6KB1, TGFBR2, PDE4D, MPO, HMGCR, SIRT3, CYP1A1, AKT1, PCK1, APAF1, NQO1, GSS
5	GO:0051092 ~ positive regulation of NF‐κ B transcription factor activity	13	1.34E‐04	PRKCI, RNF31, RPS6KA5, S100A9, RHEBL1, HSPA1A, AR, ALK, PRKCQ, PRKCB, SPHK1, CAMK2A, KRAS
6	GO:0030574 ~ collagen catabolic process	8	0.001089192	CTSS, MMP12, MMP13, MMP16, MMP3, MMP7, 4APAF1, MMP9
7	GO:0042752 ~ regulation of circadian rhythm	7	0.001362838	TOP2A, PRKAA2, EZH2, PPARA, MAPK8, PPARG, MAPK10
8	GO:0042572 ~ retinol metabolic process	5	0.006424243	CYP1B1, TTR, AKR1C3, RBP4, RBP1
9	GO:0007623 ~ circadian rhythm	7	0.011391307	RORC, NTRK2, GSK3B, TYMS, F7, NAMPT, EGFR

In addition, we analyzed the biological process (BP), molecular function (MF), and cellular component (CC) terms related to the enriched GO pathways. Among the BP, MF, and CC categories, the top 20 terms with significantly enriched rankings are displayed visually (Figure 2D–F). The main BP terms were positive regulation of response to external stimulus, response to extracellular stimulus, protein heterodimerisation activity, and positive regulation of cell adhesion; the main MF terms were protein kinase activity, protein tyrosine kinase activity, and protein serine/threonine/tyrosine kinase activity; and the main CC terms were focal adhesion, cell–cell junction, perinuclear region of cytoplasm, and extracellular matrix.

#### 3.2.3 Orthogonal Experimental Design and Results

As described in section 2.2.4, based on the results of the above single‐factor experiments, an orthogonal 2‐factor 2‐level experiment, namely, the liquid‐ingredient ratio (A) and ultrasonication time (B), was designed, and a total of 4 test points were analyzed; the results are shown in Table 7. Based on the orthogonal experiments, the optimal extraction process of the eight medicinal substances from the tailored WAW formula was obtained, as shown in the following table:

**TABLE 7:** Optimal extraction process of the 8 medicinal substances from the tailored WAW formula.

Medicinal substances	Reagents	Liquid–liquid ratio	Ultrasonic time	Number of extractions	Extraction temperature
*D. polystachya*	30% 1,3‐butanediol	1:30	70 min	2	50°C
*C. chinensis*		1:20	70 min	2	
*C. officinalis*		1:20	80 min	3	
*S. chinensis*		1:20	80 min	2	
*E. ulmoides*		1:20	60 min	3	
*A. bidentata*		1:30	50 min	3	
*P. Cocos*		1:30	70 min	3	
*A. orientalis*		1:30	70 min	3	

#### 3.2.4 Adjustment of the Compounding Ratio of the Tailored WAW Formula

The results of network pharmacology revealed that among the top 10 compounds ranked according to degree, the single herb hyssop had many effective compounds, so the proportion of hyssop was greater than that of the original formula.

The results of in vitro efficacy experiments of single herbs revealed that at the 2% treatment concentration, compared with the control group, *D. polystachya* , *C. officinalis* , *P. Cocos* , and *A. bidentata* had greater effects on promoting collagen synthesis, of which *D. polystachya* had the greatest effect (158.28%), and all of them inhibited tyrosinase activity, NO production, and melanin production. Among them, *S. chinensis* had the greatest ability to inhibit tyrosinase activity and melanogenesis (47.01% and 75.84%, respectively). *C. officinalis* and *A. bidentata* still promoted collagen at the 1% treatment concentration. Therefore, the proportion of herbs was greater than that of the original formula. *E. ulmoides* and *A. orientalis* did not clearly promote collagen production or inhibit tyrosinase activity, and as adjuvants and enablers, the samples were also darker in color, and the dosages were poorer than those of the other herbs.

Thus, by synthesizing the principles of TCM formulation with the results of network pharmacology and in vitro efficacy experiments, the combinations and compounding ratios of the single herbs of the tailored WAW formula were rearranged, as shown in Table 8.

**TABLE 8:** Three different compounding ratio formulas.

	Formula (M1)	Formula (M2)	Formula (M3)
Sovereign medicines	*E. ulmoides* : 9.0 mL	*D. polystachya* : 7.5 mL	*D. polystachya* : 9.0 mL
	*D. polystachya* : 7.5 mL	*C. chinensis* : 7.5 mL	*C. chinensis* : 6.0 mL
Minister medicines	*C. chinensis* : 6.0 mL	*E. ulmoides* : 7.5 mL	*C. officinalis* : 9.0 mL
	*C. officinalis* : 6.0 mL	*C. officinalis* : 7.5 mL	*S. chinensis* : 9.0 mL
Assistant medicines	*S. chinensis* : 7.5 mL	*S. chinensis* : 7.5 mL	*E. ulmoides* : 4.5 mL
	*A. bidentata* : 6.0 mL	*A. bidentata* : 7.5 mL	*A. bidentata* : 9.0 mL
Courier medicines	*A. orientalis* : 6.0 mL	*A. orientalis* : 7.5 mL	*P. cocos* : 9.0 mL
	*P. Cocos* : 6.0 mL	*P. cocos* : 7.5 mL	*A. orientalis* : 6.0 mL

**FIGURE 3:**
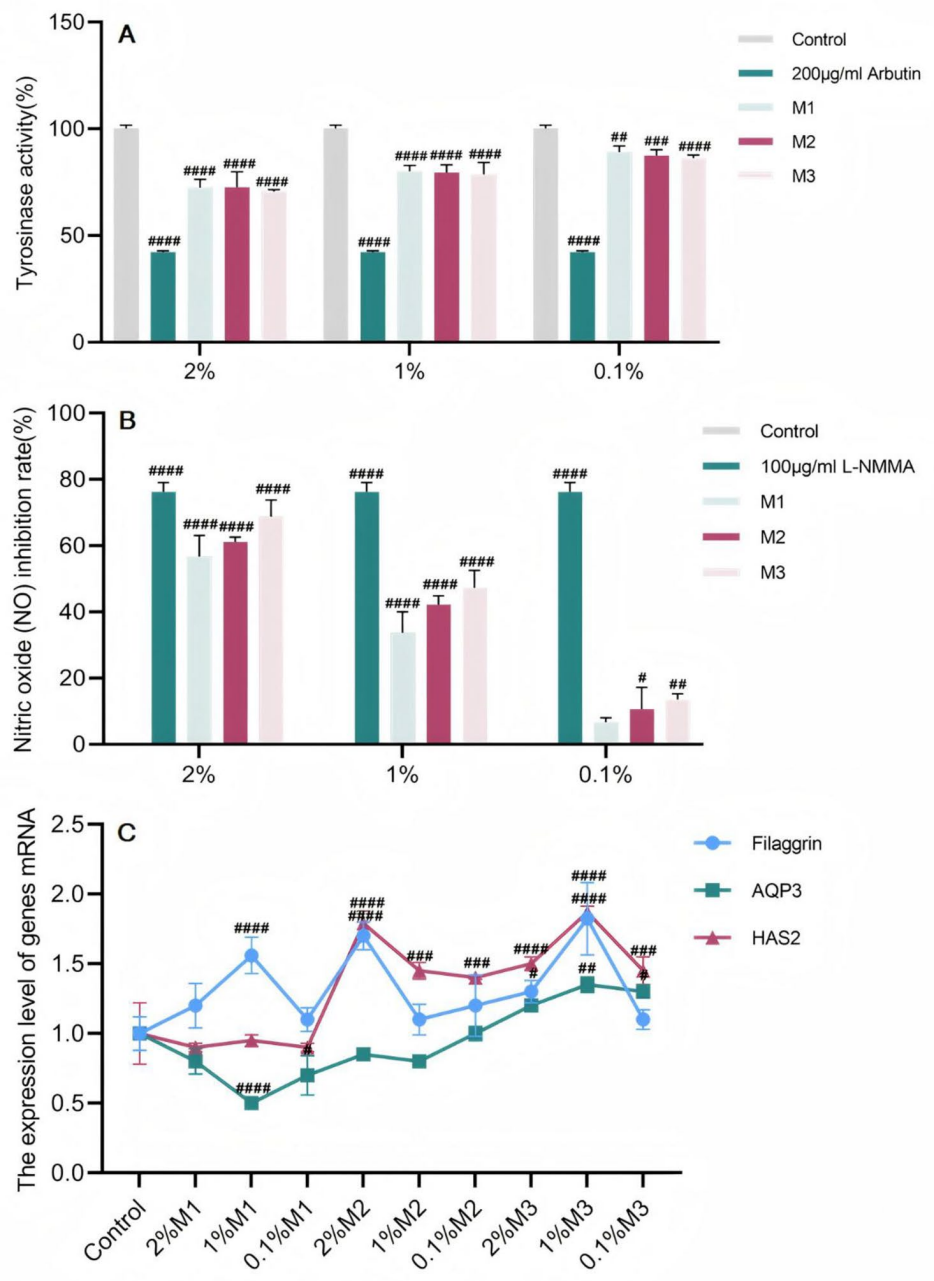
In vitro efficacy determination of three different compounding ratios. (A) Results of tyrosinase activity; (B) Results of no inhibition; (C) Results of filaggrin, AQP3, and HAS2 mRNA expression. (The results are expressed as the means ± SD. Two‐way ANOVA was performed to determine statistical significance. #*P* < 0.05, ##*P* < 0.01, ###*P* < 0.001 and ####*P* < 0.0001 compared to the control group).

Screening of the three ratios and identification of the chemical compositionIn summary, compounding should be carried out according to the above three different ratios, and parameters such as tyrosinase activity, NO production, and moisturizing genes should be measured afterwards. The results of the experiments (Figure 3A–C) revealed that M1, M2, and M3 inhibited tyrosinase activity and NO production at 2%, 1%, and 0.1% treatment concentrations, respectively. The ability of M3 at a 1% concentration to increase filaggrin mRNA expression was 1.823 times greater than that of the control group, and HAS2 mRNA expression was 1.871 times greater than that of the control group. Therefore, M3, which has the best overall effect on skin improvement, was chosen as the formula for the subsequent verification of the cellular‐level aging effect.

**FIGURE 4:**
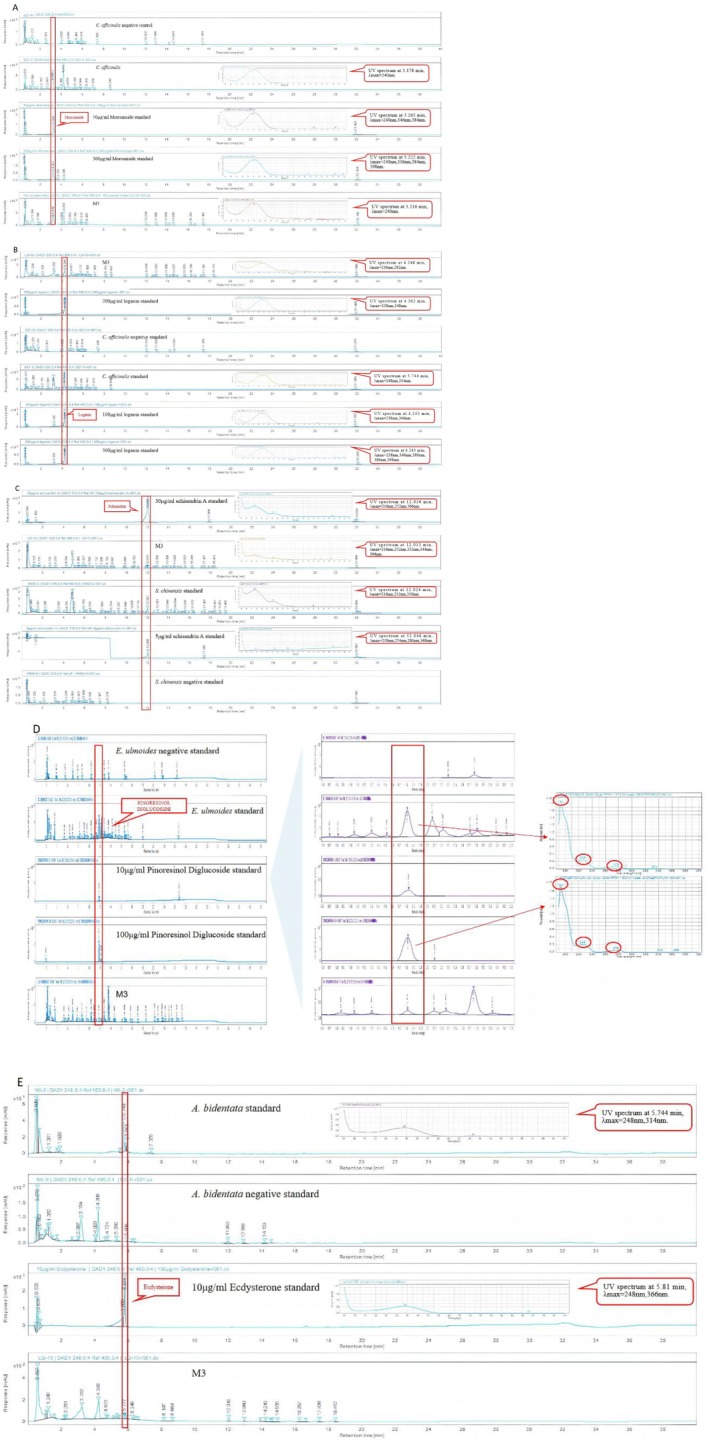
Results of M3 Component Analysis.

After the extraction process of the formula was optimized with reference to the results of network pharmacology and the optimization of the extraction process of the single herbs, the formula quality was investigated using HPLC to evaluate the characteristic components of the single herbs by retention time and ultraviolet spectrograms. The key evaluated components included morroniside, loganin, schisandrin A, pinoresinol diglucoside, and β‐ecdysterone (Figure 4).

### 3.3 Validation of the Efficacy of the Tailored WAW Formula in Delaying Aging at the Cellular Level and Exploration of the Mechanism

#### 3.3.1 Modeling Results

**FIGURE 5:**
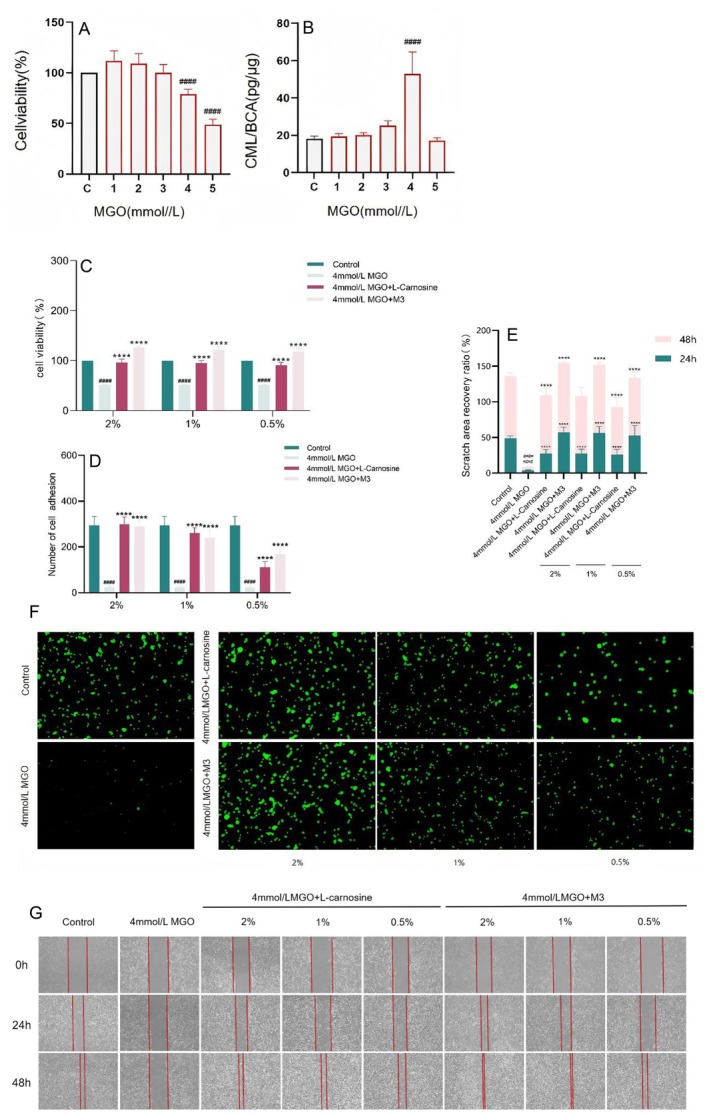
Establishment of a model of senescence injury in MGO‐cultured fibroblasts with high expression of CML and M3 ameliorates impaired cell behavioral function caused by the action of MGO on fibroblasts. (A) Effect of MGO on the survival rate of fibroblasts; (B) Effect of MGO on CML expression in fibroblasts; (C) Examination of cell proliferation ability results; (D, F) Examination of cell adhesion ability; (E, G) Examination of cell migration ability within 48 h. (The results are expressed as the means ± SD. One‐way ANOVA and Two‐way ANOVA was performed to determine statistical significance. ####*P* < 0.0001 compared to the control group. ****P < 0.0001 compared to the MGO model group).

In accordance with previous laboratory experience, a cell aging model stimulated with MGO and high AGE expression was established in human skin fibroblasts. The results revealed that 4 mM MGO stimulated fibroblasts for 48 h, and the cell survival rate was 79.25% (Figure 5A). The highest expression level of CML was 53.04 pg/μg, which significantly differed from that of the normal group (18.10 pg/μg) (Figure 5B). Therefore, 4 mM MGO stimulation of fibroblasts for 48 h was selected as the model condition.

#### 3.3.2 Results of Detecting Cellular Dysfunction Behavior

The toxicity of M3 to fibroblasts at each concentration was examined using the CCK‐8 method, and the results are shown in Figure 1A. M3 was nontoxic to cell concentrations ranging from 2% to 0.5%. Therefore, 2%, 1%, and 0.5% were selected for subsequent experiments.

The cell proliferation ability associated with damage to cellular aging was assessed using the CCK8 assay. As shown in Figure 5C, M3 effectively restored the MGO‐induced proliferation of damaged cells at concentrations of 2%, 1%, and 0.5%.

A fluorescence assay revealed that the cell adhesion capacity was associated with cellular senescence injury and that M3 ameliorated MGO‐induced senescence injury‐related cell adhesion capacity (Figure 5D,F). Compared with 2% and 1% M3, 0.5% M3 had a weaker ability to restore the adhesion of MGO‐injured fibroblasts.

A scratch assay was used to investigate the ability of M3 to improve the migration ability of MGO‐induced senescent cells at 24 h and 48 h, and the effect became more significant with increasing time (Figure 5E,G). At the 24 h modeling time range, 2% and 1% concentrations of M3 restored the migration ability of MGO‐damaged cells, and both achieved more than 50% recovery, which significantly differed from that of the MGO model group (3.51%). At 48 h, the recovery effect of 2% and 1% M3 reached over 95%, whereas the effect of 0.5% M3 on restoring the migration ability of MGO‐damaged cells reached 80% compared with that of the MGO model group (4.86%).

#### 3.3.3 Results of the ECM Protein Component Content Assay

**FIGURE 6:**
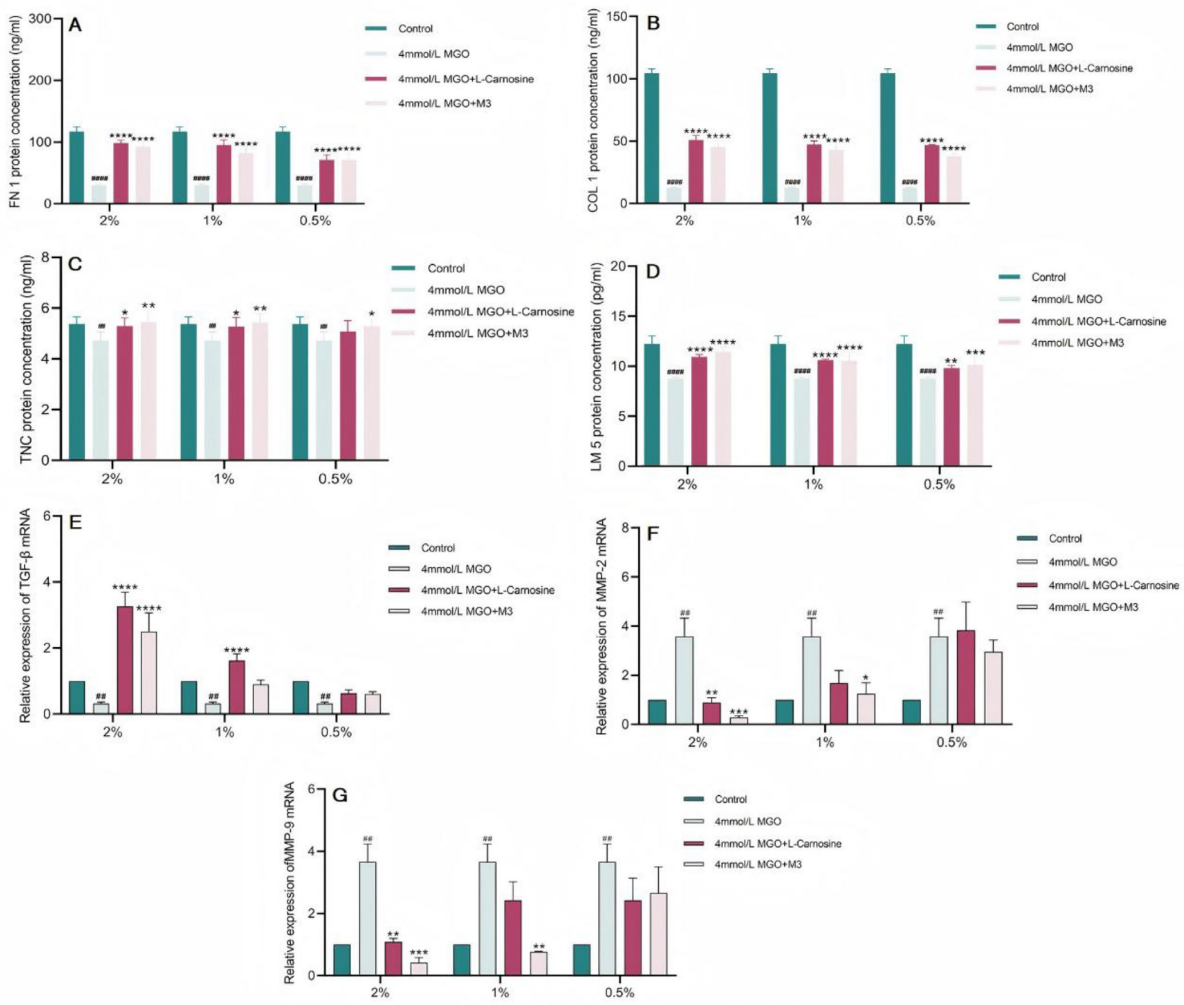
M3 improves the imbalance of protein composition in the ECM and regulates the protein production and degradation pathway of the ECM of fibroblasts induced by MGO. (A–D) Results of ELISA for FN1, COL1, TNC, and LM5 protein content; (E–G) Results of TGF‐β1, MMP‐2, and MMP‐9 mRNA expression. (The results are expressed as the means ± SD. Two‐way ANOVA was performed to determine statistical significance. ##*P* < 0.01 and ####*P* < 0.0001 compared to the control group. *P < 0.05, **P < 0.01, ***P < 0.001 and ****P < 0.0001 compared to the MGO group).

The ECM is a complex noncellular network mainly composed of fibroblasts, proteoglycans, hyaluronic acid, adhesion glycoproteins (fibronectin and laminin) and fibronectin (collagen), as well as growth factors and cytokines [8]. Among these proteins, laminin regulates cell adhesion, growth, and differentiation [9], and laminin‐5 supports cell adhesion and migration more efficiently than other laminin proteins do, and increased expression of laminin‐5 rejuvenates aging skin [10]. The multimodular ECM protein tenascin C (TNC) is not an obligatory structural component of the ECM but binds to structural proteins in the ECM and to cell surface receptors [11, 12]. The binding of TNC to these receptors activates downstream pathways and affects functions such as cell proliferation, adhesion, and migration [13, 14]. ECM glycation is manifested by increased skin stiffness and decreased elasticity, as well as the induction of fibroblast senescence and apoptosis [15]. AGEs (CML) regulate the expression of ECM proteins, altering the expression of enzymes responsible for their degradation and inhibiting pathway‐synthesized proteins [16]. M3 at 2%, 1%, and 0.5% significantly restored the ability of MGO‐injured fibroblasts to synthesize COL1, FN1, TNC, and LM5, all of which were significantly different from those of the MGO model group (Figure 6A–D). The ability of 0.5% M3 to restore the ability of MGO‐injured fibroblasts to synthesize TNC and LM5 was weaker than that of 2% and 1% M3.

#### 3.3.4 Results of the ECM Protein Component Production and Degradation Pathway Assays

The accumulation of functional abnormalities in aging dermal fibroblasts leads to increased degradation and decreased synthesis of the ECM [17, 18]. A decrease in the number of fibroblasts increases alterations and the degradation of the ECM, which manifests as progressed dermal thinning, increased wrinkling, and a loss of elasticity [19]. This degradation process is mediated mainly by the matrix metalloproteinase family (metalloproteinases, MMPs). ECM synthesis and degradation in dermal fibroblasts are regulated mainly by the TGF‐β signaling pathway, which is able to (i) promote the expression of ECM genes and (ii) downregulate MMPs to promote ECM protein synthesis [20, 21]. The results revealed that 2% M3 significantly increased the expression of the TGF‐β1 mRNA in MGO‐damaged fibroblasts, whereas the other concentrations did not restore TGF‐β1 mRNA expression. M3 at a concentration of 2% inhibited MMP‐2 mRNA expression in MGO‐damaged fibroblasts, whereas 1% M3 minimally inhibited MMP‐2 and MMP‐9 mRNA expression, and 0.5% M3 did not inhibit the ability of MGO‐damaged fibroblasts (Figure 6E–G).

**FIGURE 7:**
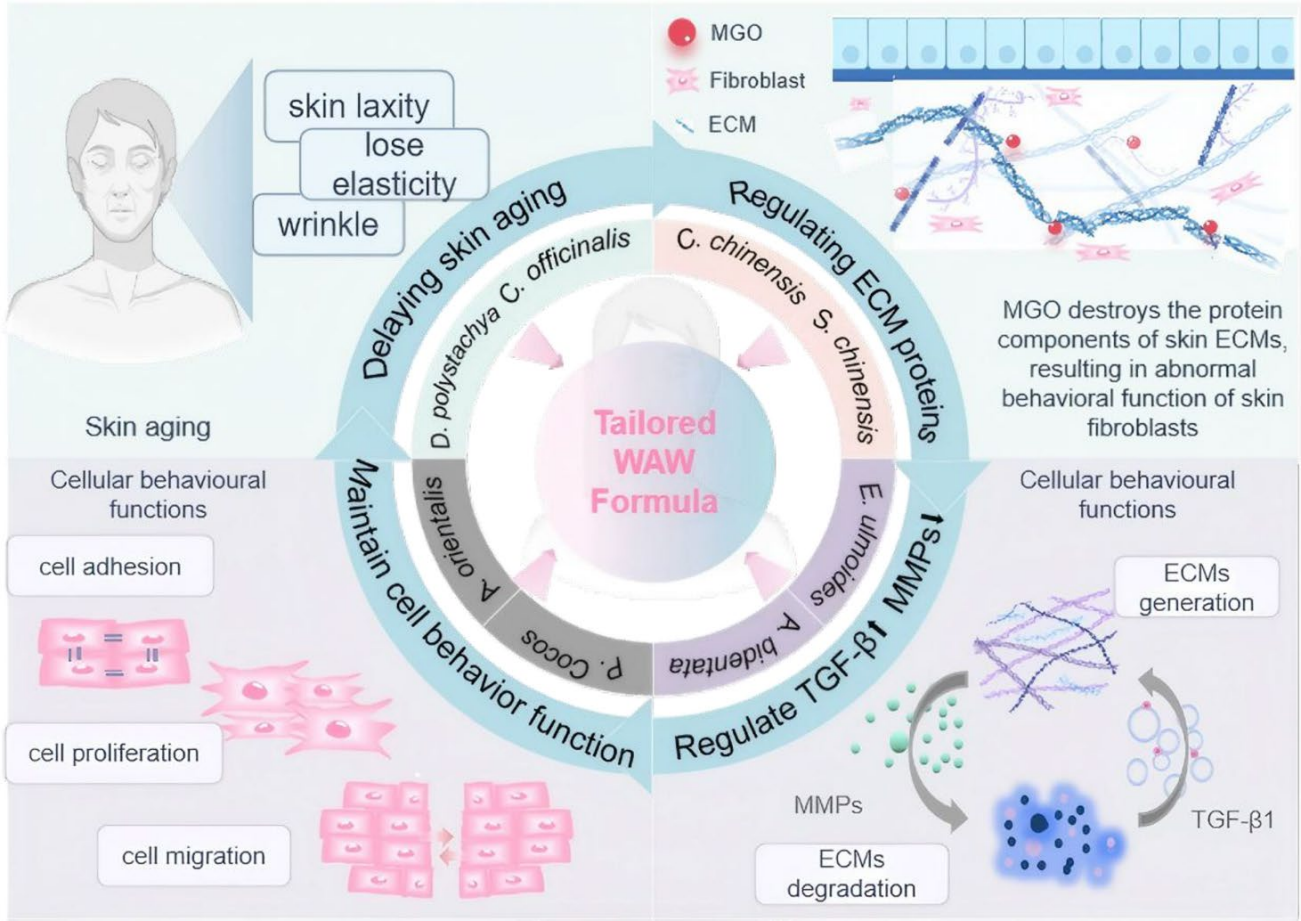
Diagram of the mechanism by which the tailored WAW formula ameliorates MGO‐induced senescence damage in fibroblasts.

In summary, M3 was better at restoring the proliferation ability and migration ability of damaged cells and restoring the adhesion ability of damaged cells. Moreover, its ability to restore the expression of the ECM protein components type I collagen/COL1 and fibronectin/FN1 was greater, and its ability to restore the expression of the tendon proteins C/TNC and laminin 5/LM 5 was weaker. M3 promoted ECM protein production mainly by upregulating TGF‐β. M3 also inhibited the degradation of ECM protein components by downregulating MMP‐2 and MMP‐9, thereby restoring the functions of migration, adhesion, and proliferation of damaged cells; slowing cellular aging; and exerting firming and anti‐wrinkle effects on the skin (Figure 7).

## 4 Discussion

Traditional Chinese medicine formulas include a variety of effective components that impact the body through integrated regulation of multiple systems, multiple pathways, and multiple targets. With the holistic concept of compounding medication and other advantages and characteristics of the raw materials of Chinese herbal medicine, the active ingredients are effective for external use and can provide comprehensive skincare in line with consumers' preferences for natural products and their health needs. TCM formulas composed of two or more herbs can often result in better curative efficacy and fewer side effects than those composed of single herbs [22, 23]. In recent years, traditional Chinese medicine compounds have shown excellent results in anti‐skin aging treatment [24]. Wang Chunyu et al. [25] reported that Xiao Chaihu Tang had a significant protective effect on H_2_O_2_‐induced oxidative stress injury in human skin fibroblasts, which may be related to the delay of skin aging and the mechanism involving a decrease in the expression of the NF‐κB pathway. Ma Zhuofei et al. [26] reported that Yupingfeng San had a protective effect against photoageing in human skin fibroblasts.

At present, some progress has been made in Chinese medicine research on skin aging, but many challenges remain in the field. First, most traditional Chinese medicines are internal formulas, and there are relatively few studies on the external use of formulas to slow skin aging. Additionally, relatively few studies exist on the external use of formulas to slow skin aging, and the existing studies may not comply with modern medical theories or safety concerns regarding the external use process. It is important to combine the results of modern tests with addition, subtraction and dosage adjustments to improve the efficacy of Chinese medicine compounding and to lay the foundation for innovative Chinese medicine research. Second, the specific mechanism of action of ancient medicines used for skin has not been thoroughly studied in modern science, and there are uncertainties in their compounding relationships, formulas and mechanisms of action. Clear pharmacological support is necessary to provide sufficient persuasive power to support the use of these prescriptions in the scientific and medical fields. However, methods to make herbal formulas safer and more effective at improving the skin have been widely recognized in modern medicine. Therefore, in this study, “Wan An Wan” was used as the source of the formula, which was modified, and the compounding ratio was adjusted. Network pharmacology and HPLC were subsequently used to explore the main active ingredients of the tailored WAW formula and their molecular mechanisms in delaying aging. To verify the bioinformatics results, cell‐level experiments were conducted to evaluate the pharmacological effects of the tailored WAW formula.

“Wan An Wan” was first recorded in “Unparalleled *Dioscorea* Pills,” Tang—“Preparing the Emergency Thousand Golden Essentials”—Volume 19, as a prescription for the “King of Medicine,” “Sun Simiao,” to make one's “body moisturized, lips and mouth red, hands and feet warmed,” and the face has a light and happy recent research has reported that the main ingredients of “Wan An Wan” are *C. chinensis* , *C. officinalis* , *S. chinensis* , *A. orientalis* and *P. cocos* . These ingredients are rich in flavonoids, polysaccharides, lignans, and steroids, which have antioxidant, anti‐inflammatory, whitening, aging‐delaying, and pigmentation‐treating effects, indicating their potential for skin improvement. Therefore, the present study combined an analysis of drug interactions and the law of compounding to customize “Wan An Wan” into the following formula: the sovereign medicines *D. polystachya* and *C. chinensis* ; minister medicines *C. officinalis* and *S. chinensis* ; assistant medicines *E. ulmoides* and *A. bidentata* ; and courier medicines *P. cocos* and *A. orientalis* .

According to the drug‐active ingredient‐target network, the main active ingredients of the tailored WAW formula were cycloartenoids, lignans, polysaccharides, flavonoids, etc. The extraction process was optimized, and after the optimal combination ratio of the formulas was determined, the chemical constituents were identified by HPLC. There were 412 skin‐related active ingredients in the WAW formula, indicating that the formula exerts pharmacological effects on skin aging through multiple targets. KEGG and GO pathway enrichment analyses revealed that the WAW formula affected the skin through a total of 21 pathways and was enriched in the MAPK signaling pathway, which is related to increased skin aging, collagen decomposition and metabolism, the extracellular matrix (ECM) and the skin aging process. Among them, matrix metalloproteinases (MMPs) and TGF‐β may be genes related to the active ingredient of the tailored WAW formula to slow the aging process.

One of the characteristics of skin aging is alterations in the structure and function of the dermis, where fibroblasts produce dermal ECM and maintain its homeostasis. During the aging process, MMPs gradually degrade the ECM, disrupting the interaction between fibroblasts and the ECM and affecting cellular phenotype changes (such as proliferation, migration, differentiation, and apoptosis), thereby altering the structure and function of the dermis. TGF‐β coordinates ECM production and promotes COL1 synthesis in aging fibroblasts, thereby maintaining the structural stability of the ECM and delaying aging. In summary, based on cellular‐level experiments, the tailored WAW formula acts on skin fibroblasts, modulating the upregulation of TGF‐β1 and downregulation of MMP‐2/MMP‐9, regulating the production and degradation of ECM proteins, and adjusting cell behaviors such as migration, adhesion, and proliferation, thus normalizing the skin and maintaining its health.

Owing to their unique cultural heritage and scientific value, skincare products based on Chinese medicine have earned a place in the modern skincare market. In the future, with advances in science and technology and changes in market demand, the research, development, and application of Chinese medicine skin care products will become more diversified and internationalized.

## 5 Conclusion

In this study, we constructed an overall research and development strategy to effectively tailor the formula and adjust the compounding ratio of the imperial prescription “Wan An Wan” to allow the resulting findings to be better integrated with modern research on improving skin aging. After a preliminary investigation of the in vitro efficacy of the single medicinal substances in the formula, we used the network pharmacology method to explore the mechanism by which the tailored WAW formula slows skin aging. We determined that iridoids, lignans, polysaccharides, and flavonoids are the likely active ingredients. Moreover, the mechanism of action may involve the production and degradation of ECM proteins through the promotion of the activation of signaling pathways, such as the TGF‐β pathway, and the inhibition of MMPs and other targets. At the cellular level, the tailored WAW formula enhanced cell proliferation, migration and adhesion. The results of this study provide a theoretical basis for the subsequent development of herbal formulas for skin health applications and further developments and innovations in TCM cosmetic science.

## Author Contributions

**Yingying Lin:** writing – original draft and visualization. **Yunhee Chang:** writing – review and editing. **Yoojae Maeng, Xiaoxing Liu, Xinke Liu:** data curation. **Hong Meng:** resources. **Fan Yi:** conceptualization, funding acquisition, and project administration.

## Conflicts of Interest

The authors declare no conflicts of interest.

## Data Availability

The data that support the findings of this study are available from the corresponding author upon reasonable request.
